# Advances in hydrogel-based materials for breast cancer bone metastasis: from targeted drug delivery to bone microenvironment remodeling

**DOI:** 10.3389/fphar.2025.1627883

**Published:** 2025-06-24

**Authors:** Jiayi Chen, Jun Ma, Zhuoming Xu, Huanhuan Luo, Chenhong Qian

**Affiliations:** Jiaxing Key Laboratory of Basic Research and Clinical Translation on Orthopedic Biomaterials, Department of Orthopaedics, The Second Affiliated Hospital of Jiaxing University, Jiaxing, China

**Keywords:** breast cancer, bone metastasis, drug delivery, multifunctional platform, bone regeneration

## Abstract

Breast cancer has become the most common malignant tumor in women around the world, and bones are the most common part of all metastatic breast cancers. Breast cancer bone metastasis (BCBM) is the main cause of death in patients with advanced breast cancer. It is still mainly clinically palliative treatment, with problems such as systemic toxicity, low target specificity, and insufficient bone repair. Therefore, there is an urgent need to develop new therapeutic strategies to overcome these challenges. This review summarizes recent advances and innovative applications of smart hydrogel-based delivery systems for breast cancer bone metastasis, highlighting their significant potential in gene delivery and immune microenvironment remodeling. Current limitations and future research directions are also discussed.

## 1 Introduction

Based on global cancer statistics, breast cancer (BC) has emerged as the malignant tumor with the highest incidence rate worldwide (Li Q. et al., 2024). Epidemiological prediction models suggest that the incidence and mortality of the disease will continue to show a stable upward trend in the next few decades. The 2022 Global Cancer Statistics Report further pointed out that breast cancer ranks second in the female cancer spectrum with 2.3 million new cases (accounting for 11.6% of the total number of malignant tumors in women). At the same time, as one of the main causes of cancer-related deaths worldwide, its disease burden continues to increase (Bray et al., 2024; Giaquinto et al., 2024; Liao, 2025). In recent years, significant progress has been made in the field of breast cancer diagnosis and treatment: the innovation of diagnostic methods based on imaging omics and multimodal imaging technology has significantly improved the early detection rate, and the combination of the establishment of a standardized screening system and the combination of refined physical examination technology has made a substantial breakthrough in the timeliness of clinical diagnosis. Constructing an interdisciplinary collaborative network promotes surgical optimization, innovation of targeted drug delivery systems, and the formulation of individualized and precise treatment plans. This breakthrough progress not only reconstructs the clinical diagnosis and treatment paradigm of breast cancer but also significantly improves patients’ survival prognosis and quality of life through the accumulation of evidence-based medical evidence (Bottosso et al., 2024; Park et al., 2024; Zhang-Petersen et al., 2024).

Despite advances in diagnostics and therapies, BCBM management remains clinically challenging (Pantel and Hayes, 2018). Metastatic dissemination represents the leading cause of breast cancer-specific mortality. It mainly includes the skeletal system, lung parenchyma, liver, and central nervous system. Skeletal-related events (SREs) caused by bone metastasis are particularly worthy of attention. Breast cancer has become the most common malignant tumor in women around the world, and bones are the most common part of all metastatic breast cancers. Bone metastasis is one of the major complications of advanced breast cancer. Its pathological features are mainly osteolytic bone destruction, which usually leads to pain, pathological fracture, and hypercalcemia, which seriously affects the quality of life and prognosis of patients (Verbeeck, 2004; Li et al., 2023). Currently, the main treatment options for BCBM aim to extend patient lives and relieve related symptoms. Currently, most clinical treatments are palliative, focusing mainly on pain management, reducing the risk of SREs, and suppressing tumor progression (Oldenburger et al., 2022). Current treatments for BCBM mainly include local treatment (surgery and radiotherapy) and systemic treatment (chemotherapy, targeted treatment, and bone modifiers to reduce bone destruction). Although the existing treatment methods can partially alleviate symptoms, their efficacy is limited by high systemic toxicity, low targeting efficiency, and the lack of bone repair ability (Souchon et al., 2009; Jacobs et al., 2014; Stevens and Hellig, 2022; Ait Oumghar et al., 2024). Therefore, it is imperative to urgently develop innovative treatment strategies to overcome these challenges.

**SCHEME 1:**
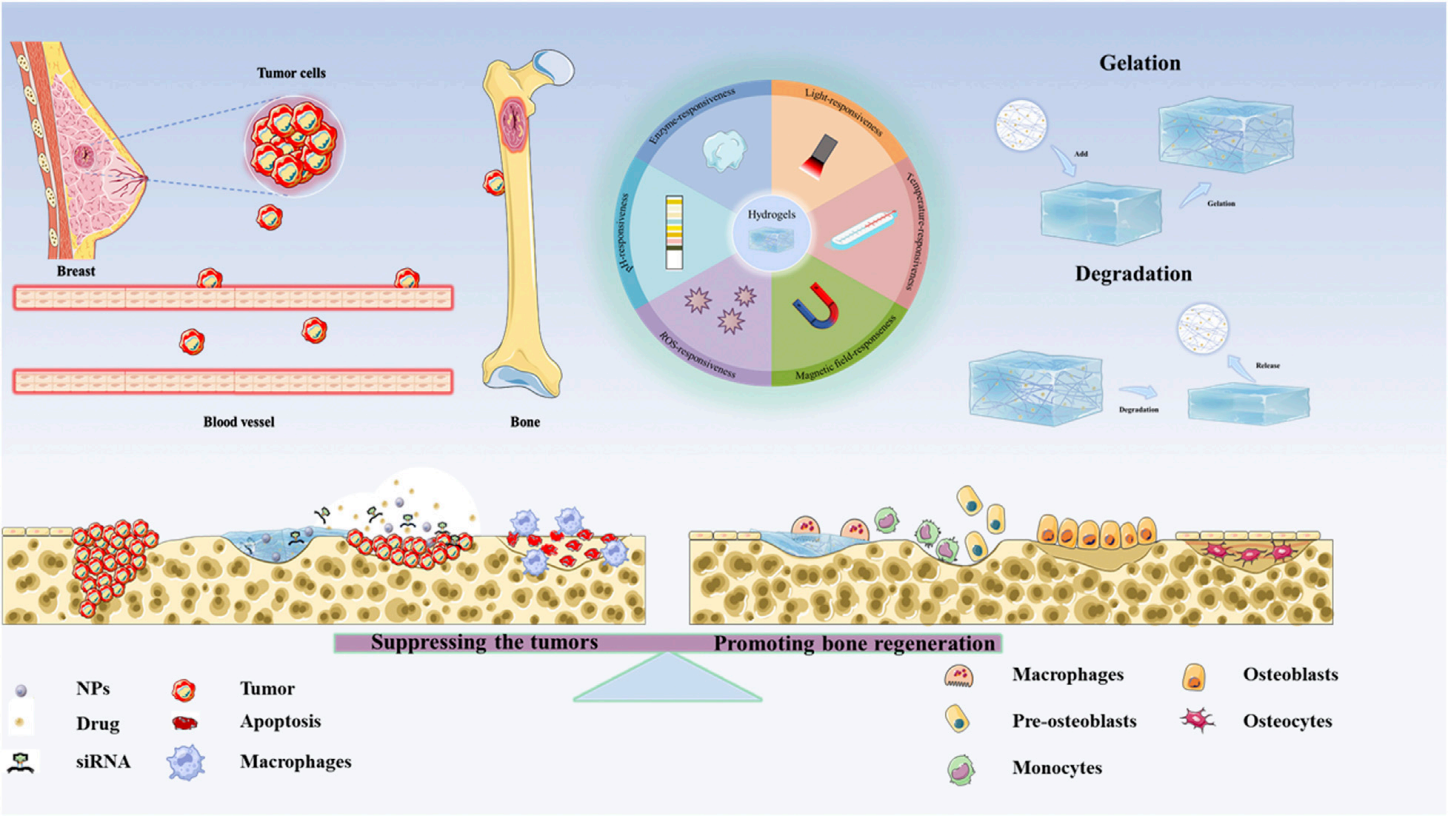
The applications of Hydrogel-based therapeutic systems for suppressing the tumors and Remodeling Bone Microenvironment in BCBM.

Recent biomaterial innovations, particularly intelligent hydrogel systems, offer novel therapeutic approaches for the treatment of BCBM. Hydrogels are three-dimensional network structures formed by cross-linked hydrophilic polymers. They have the advantages of high-water content, injectability, and biomimetic extracellular matrix characteristics. They have become ideal drug delivery carriers and tissue engineering scaffolds (Singhal et al., 2020; Bordbar-Khiabani and Gasik, 2022). Compared with traditional intravenous administration, hydrogels improve the therapeutic efficiency through the local and precise delivery of drugs and effectively reduce systemic toxicity. Critically, multifunctional engineering enables stimuli-responsive drug release targeting bone metastasis microenvironments, while modulating the immune microenvironment to disrupt the bone metastasis vicious cycle (Esmaeili et al., 2021; Xie et al., 2021; Shao et al., 2022). Despite diagnostic and therapeutic advances, significant clinical challenges persist in BCBM management, with substantial translational barriers remaining (Tian et al., 2022; Yang Q. et al., 2025). Based on the above background, this review focuses on recent advances in hydrogel-based systems for BCBM, highlighting their application potential in gene delivery and bone immune microenvironment remodeling. It serves as a theoretical reference for researchers, aiming to provide novel insights for the precision therapy of BCBM (Scheme 1).

## 2 Pathological mechanisms of BCBM

**FIGURE 1:**
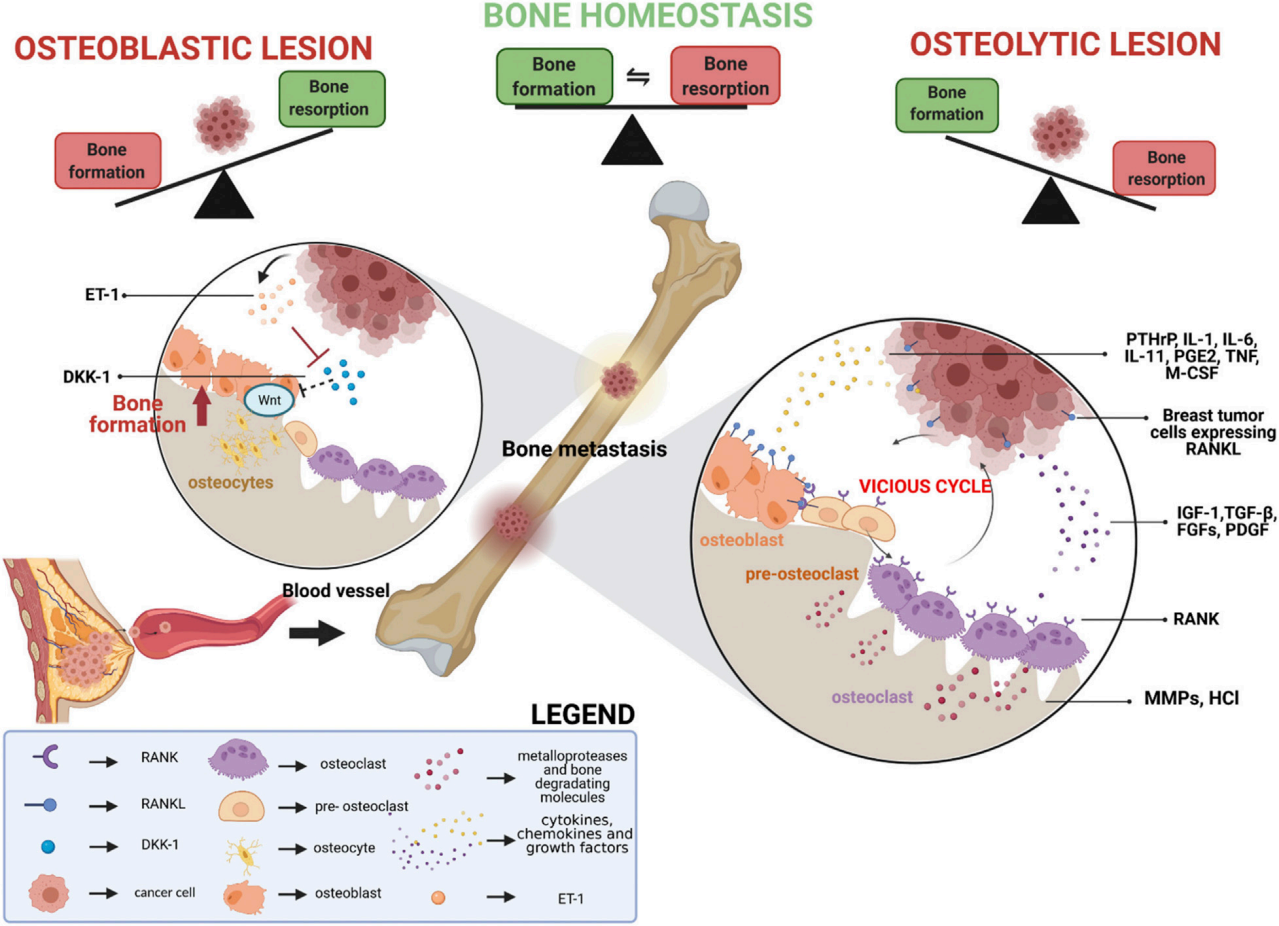
Schematic representation of the processes involved in breast cancer bone metastasis formation. Reprinted with permission from (Venetis et al., 2021).

The occurrence and development of breast cancer bone metastasis is a very complex process. Studies have shown that multiple mechanisms are involved in the initiation and progression of bone metastasis in breast cancer. In the early stage of metastasis, breast cancer cells are used for effective “sowing” of tumor cells by establishing the microenvironment before metastasis as “soil” (Wang C. et al., 2021). For example, exosomes secreted by tumor cells can specifically fuse with target organs to induce the formation of a pre-metastatic niche (Wortzel et al., 2019). Moreover, prior to metastatic establishment, primary tumors can induce pericytes (perivascular cells) to form pre-metastatic niches. During bone tissue colonization, breast cancer cells secrete lysyl oxidase (LOX) and connective tissue growth factor (CTGF), which induce extracellular matrix sclerosis and angiogenesis, thereby facilitating tumor cell proliferation and metastatic establishment. Bone metastases can be classified as osteolytic, osteoblastic, or mixed. During osteoblastic metastasis, tumor cells secrete endothelin-1 (ET-1) to suppress the expression of Dickkopf-1 (DKK1) in osteoblasts, thereby relieving the inhibition of the Wnt signaling pathway. The activation of Wnt promotes osteoblast differentiation and bone formation, while the resulting abnormal bone structures provide shelter for tumor cells. Simultaneously, tumor cells release bone morphogenetic proteins (BMPs), which activate the Smad pathway, leading to excessive activation of osteoblasts and ectopic bone formation. Following the colonization of breast cancer cells, the survival and metastasis of tumor cells depend on their interactions with the bone microenvironment. Breast cancer cells stimulate osteoblasts to release receptor activator of nuclear factor kappa-B ligand (RANKL) through the secretion of parathyroid hormone-related protein (PTHrP), thereby activating the NF-κB and MAPK pathways to promote osteoclast differentiation and activation (Ohshiba et al., 2003; Wu et al., 2020; Kim et al., 2022). This process leads to the occurrence of osteolytic bone destruction. This bone destruction is not a unidirectional effect but rather triggers a complex “vicious cycle.” (Venetis et al., 2021; Bonni et al., 2024). During the degradation of the bone matrix, numerous bioactive molecules (such as growth factors like TGF-β, IGF-1, FGF, etc.) are released into the local microenvironment (Figure 1). These factors, in turn, further stimulate the proliferation, invasion, and PTHrP secretion of breast cancer cells, thereby exacerbating tumor progression and bone tissue destruction (Johnson et al., 2022; Kim et al., 2023; Duong et al., 2025).

## 3 Immune microenvironment of BCBM

The self-perpetuating cycle in breast cancer bone metastasis not only drives osteolytic destruction and tumor proliferation but actively establishes an immunosuppressive “triple-barrier” microenvironment—where physical confinement, cellular suppression, and molecular blockade collaboratively generate an immunologically cold niche (Coleman et al., 2020). During BCBM, aberrant osteoblast activation drives pathological collagen deposition via the SCUBE2-SHH signaling axis, impeding T-cell infiltration. Concurrently, engagement of the inhibitory receptor LAIR1 on immune cells suppresses NK and lymphocyte functions (Wu et al., 2023). Furthermore, tumor-mediated lactate efflux through MCT4 acidifies the microenvironment, directly inhibiting pyruvate kinase M2 (PKM2) activity in T cells (Yuan et al., 2021). This metabolic disruption reduces ATP synthesis and IFN-γ secretion. Lactate also activates GPR81 to upregulate PD-L1 on tumor cells, facilitating immune evasion (Guo et al., 2023; Lundø et al., 2023; Okui et al., 2023).

In the bone microenvironment, tumor cells can exploit various immune cells to achieve immune escape and promote the spread and growth of cancer cells in bone. For example, breast cancer cells can secrete transforming growth factor-β (TGF-β) and prostaglandin E2 (PGE2) to induce the differentiation of dendritic cells (DCs) into immune-tolerant DCs, thereby inhibiting the immune response of T cells (Zhou Z. et al., 2024). Meanwhile, granulocyte-macrophage colony-stimulating factor (GM-CSF), granulocyte colony-stimulating factor (G-CSF) and interleukin-6 (IL-6) produced by tumor cells promote the generation of myeloid-derived suppressor cells (MDSCs). MDSCs inhibit the activation and infiltration of intratumoral T cells by enhancing arginase-1 (Arg-1)-mediated arginine depletion (Li et al., 2018; Liu H. et al., 2023). Concurrently, M2-polarized TAMs secrete IL-10/TGF-β to drive Treg expansion while directly suppressing T-cell cytotoxicity via PD-L1/CTLA-4 (Santoni et al., 2018; Ren et al., 2023; Garcia-Santillan et al., 2024). Furthermore, bone marrow-derived neutrophils form neutrophil extracellular traps (NETs) that facilitate metastasis by entrapping disseminated tumor cells and suppressing cytotoxic lymphocytes, accelerating metastatic outgrowth (Garner et al., 2025; Zhang L. et al., 2025).

Abnormal signal pathway transduction further exacerbates immune escape. For instance, osteoclasts highly express CD155, which binds to the TIGIT receptor on T cells and inhibits the CD226 co-stimulatory signal, leading to a significant decline in the proliferative capacity of T cells (Gao et al., 2017). Myeloid-derived suppressor cells (MDSCs) secrete IL-1β to further expand the TIGIT^+^ T cell population, forming a “TIGIT-IL-1β-MDSC” positive feedback loop (Feng et al., 2024; Zhang Z. et al., 2024). Moreover, indoleamine 2,3 - dioxygenase 1 (IDO1) mediates the conversion of tryptophan to kynurenine, activating the aryl hydrocarbon receptor (AhR) pathway to induce the differentiation and expansion of regulatory T cells (Solvay et al., 2023). The lipid metabolism gene suppressor of cytokine signaling 3 (SOCS3) blocks the STAT3 signal, resulting in impaired activation of CD8^+^ T cells and further intensifying immune suppression (Gao et al., 2023).

## 4 Clinical treatments for BCBM

### 4.1 Surgery

Bone metastasis of breast cancer frequently induces skeletal-related events (SREs), including debilitating bone pain, pathological fractures, and spinal cord compression, which collectively and profoundly impair patients’ quality of life. Current clinical evidence suggests that timely surgical intervention serves as a critical therapeutic strategy, offering dual benefits of symptomatic relief and potential improvement in survival outcomes through local disease control (Weitao et al., 2022). With the advancement of medical technology, various image-guided minimally invasive interventional treatments have made significant progress. These techniques include ablation therapies (such as radiofrequency ablation, microwave ablation, laser ablation, and high-intensity focused ultrasound), minimally invasive endoscopic surgery, and brachytherapy such as radioactive seed implantation. These approaches not only demonstrate favorable therapeutic efficacy but also minimize damage to surrounding tissues and reduce postoperative complications, thereby facilitating accelerated patient recovery (Smith, 2011). However, although surgical intervention holds significant clinical value in alleviating SREs, its application still faces numerous challenges and limitations. First, the choice of surgical indications is highly dependent on patient survival prediction and assessment of physical status. Secondly, open surgery is highly traumatic and has a long recovery period, which can easily lead to complications such as nerve damage, infection, and internal fixation failure. In addition, secondary intervention may be required due to tumor residue or recurrence after surgery. Although minimally invasive techniques can reduce trauma, there is insufficient evidence of long-term efficacy and limited effect on decompression and stable repair of complex anatomical areas such as the spine and pelvis (Tahara et al., 2019; Cazzato et al., 2023; Suresh et al., 2025).

### 4.2 Osteoclast-targeted therapies

Bisphosphonates, as conventional anti-resorptive agents administered intravenously, primarily exert therapeutic effects by inhibiting osteoclast activity, thereby reducing bone destruction (Coleman, 2020; 2023; D’Oronzo et al., 2021; Jayan et al., 2023). However, prolonged administration may lead to systemic toxicities (e.g., hypocalcemia, renal impairment) and severe complications such as osteonecrosis of the jaw (ONJ) and atypical femoral fractures (Marcianò et al., 2020; Matsuura et al., 2021; Wei et al., 2021). With the elucidation of the self-perpetuating cycle mechanism in the bone metastasis microenvironment, researchers have discovered more targeted and safer treatment strategies. For example, denosumab (a RANKL inhibitor) has been proven by relevant studies to not only effectively block RANKL-mediated osteoclast activation, but also reduce the toxic and side effects associated with traditional bisphosphonates (Coleman et al., 2021; Wajda et al., 2025). However, the long-term efficacy and potential resistance mechanisms associated with denosumab still require further study (Philipponnet et al., 2018). Emerging anti-resorptive agents, including cathepsin K inhibitors and the monoclonal antibody 15D11, have shown considerable promise in targeting pathological bone resorption. Notably, the cathepsin K inhibitor odanacatib demonstrated robust efficacy in suppressing bone resorption among breast cancer patients with bone metastases. However, its phase III clinical development was halted due to emerging cardiovascular safety concerns (Luo et al., 2017). By inhibiting this signaling pathway, the monoclonal antibody 15D11 targeting the Jagg1 protein can effectively block over-activation of osteoclasts induced by tumor cells, and has shown significant anti-tumor effects in preclinical models. However, further research is still needed to promote its clinical application and verify its efficacy and safety through clinical trials (Zheng et al., 2017).

### 4.3 Immunotherapy

Over the past decade, immunotherapy has achieved groundbreaking progress in the management of malignant neoplasms. Emerging evidence indicates that PD-1/PD-L1 blockade therapy suppresses osteoclastogenesis through immunomodulatory mechanisms, thereby conferring sustained therapeutic benefits including prevention of bone destruction and mitigation of cancer-induced ostealgia (Wang et al., 2020). Furthermore, multiple immune cell populations have emerged as promising therapeutic targets for metastatic cancer. Notably, dendritic cells (DCs), leveraging their professional antigen-presenting capabilities, exhibit potential for both metastasis prevention and therapeutic intervention (Harari et al., 2020). Targeting CC chemokine ligand 18 (CCL18), a soluble mediator secreted by tumor-associated macrophages (TAMs), has been shown to effectively suppress breast cancer metastasis by disrupting pro-tumoral signaling pathways. This approach highlights the potential of modulating the tumor microenvironment to inhibit disease progression (Liang et al., 2018). Additionally, the suppression of myeloid-derived suppressor cell (MDSC) functional activity, or the depletion of regulatory T cells (Tregs), has demonstrated significant efficacy in reducing tumor burden and diminishing the risk of metastasis. Nevertheless, despite immunotherapy having made significant progress in tumor metastasis, in the treatment of breast cancer bone metastases, there are still problems such as immunosuppressive microenvironment and low efficiency of bone-targeted drug delivery (Liu et al., 2021).

### 4.4 Radiation therapy

Radiation therapy effectively alleviates bone metastasis-induced pain, reduces the incidence of pathological fractures, and mitigates tumor-induced spinal cord compression (Westhoff et al., 2015). External beam radiation therapy (EBRT) is currently widely used in patients with short life expectancy or who need another radiotherapy due to worsening bone pain. Compared with EBRT, stereotactic radiotherapy (SBRT) can more accurately affect tumor foci and minimize damage to healthy tissues. For patients with bone metastases, SBRT has better clinical outcomes than traditional EBRT (Sprave et al., 2018). Radium-223 (^223^Ra), an alpha-particle-emitting radiopharmaceutical used in internal radiotherapy, exhibits selective accumulation in areas of increased osteogenic activity. The high-energy, short-range alpha radiation emitted by this radiopharmaceutical enables targeted tumor cell cytotoxicity while minimizing collateral damage to adjacent healthy tissues (Zustovich and Barsanti, 2017; Ueno et al., 2020; Rugo et al., 2024). However, although radiation therapy has certain efficacy in controlling local tumor growth, its irreversible damage to surrounding normal bone tissue limits its widespread application (Marazzi et al., 2020; Miyashita et al., 2023; Nagpal et al., 2025; Zhang Y. et al., 2025). Accumulating evidence indicates that radiotherapy may cause long-term complications, including lymphedema, cardiopulmonary toxicity, neuropathy, rib fractures, and secondary malignancies, which in turn severely affect patients’ quality of life (Truong et al., 2004).

## 5 The multidimensional advantages of hydrogel materials in treating BCBM

The rapid proliferation and invasion of breast cancer cells and the vicious cycle of the bone microenvironment remain the main reasons why the survival rate of BCBM patients has not improved for decades (Attama et al., 2022). There is an urgent need to explore new methods for treating BCBM. Hydrogels, with their unique physicochemical properties and functional designability, possess multi-dimensional advantages of inhibiting tumors, reducing bone destruction, and promoting bone repair, showing great potential in the treatment of BCBM (Shao et al., 2022; Wang et al., 2023). In this section, we will introduce and discuss the latest research and application prospects of these intelligent hydrogel systems.

### 5.1 Intelligent responsive drug controlled-release hydrogel system

Hydrogel-based drug delivery systems circumvent the dose-limiting toxicity of conventional chemotherapy by achieving localized, controlled, and sustained drug release (Kanda et al., 2021). Recent advances in hydrogel engineering have driven the evolution of these biomaterials from conventional natural/synthetic compositions to intelligent responsive systems designed for spatiotemporally precise drug delivery (Shao et al., 2022). Intelligent responsive hydrogels present novel strategies for overcoming therapeutic barriers in BCBM by enabling spatiotemporally controlled drug release through sensing tumor microenvironment (TME)-specific signals or responding to exogenous stimuli (Table 1).

**TABLE 1:** Summary of stimuli-responsive hydrogels composites in breast cancer local therapy.

Type of stimulus	Hydrogel composition	Tumor therapy strategies	Ref.
pH	Chitosan Glyceryl Monooleate Noisomes TZO (A Tamoxifen analogue)	Therapeutic drug delivery	Salem et al. (2020)
	Chitosan Polyethylene Glycol Sodium Bicarbonate Doxorubicin	Therapeutic drug delivery Regulation of tumor microenvironment pH	Ahmed et al. (2023)
ROS	Chitosan Ferulic Acid Thioketal Pazopanib AQ4N	Therapeutic drug delivery “Starvation therapy”	Chen et al. (2023)
Enzyme	Phosphorylated Tyrosine Gly-Phe-Phe-Tyr Carboxylesterase Lonidamine	Therapeutic drug delivery	Wu et al. (2021)
Enzyme Magnetic fields	Gelatin Methacryloyl Doxorubicin Fe_3_O_4_@PVP PEGDA BAPO	Therapeutic drug delivery	Tian et al. (2025)
Light Temperature	Polydopamine Collagen Silk Fibroin Thrombin Complementary Oligonucleotides	Photothermal Therapy “Nutrition deprivation strategy”	Wang et al. (2021)
Light Temperature	Agarose AIE Thioridazine DSPE-PEG	Therapeutic drug delivery Photothermal Therapy	Zhang T. et al. (2024)
Magnetic fields Temperature	Sodium Alginate Doxorubicin Fe_3_O_4_ MNPs	Therapeutic drug delivery Magneto-thermal effect	Hu et al. (2024)

#### 5.1.1 Endogenous stimulus response

**FIGURE 2:**
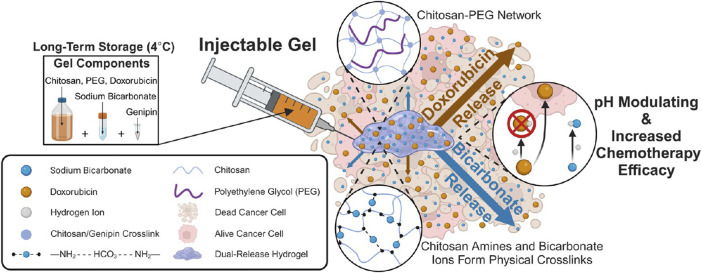
Injectable Chitosan-PEG Hydrogel for Dual Delivery of Sodium Bicarbonate and Doxorubicin for Increasing Chemotherapy Efficacy. Reprinted with permission from (Ahmed et al., 2023).

The TME exhibits pathologically acidic conditions (pH 6.5–6.8), providing an intrinsic trigger for spatiotemporally controlled drug release in pH-responsive delivery systems (Hasegawa et al., 2025). A non-ionic surfactant vesicle hydrogel system loaded with a pH-responsive triaryl-(Z)-olefin (TZO) was developed for breast cancer therapy. Under acidic tumor microenvironment conditions, the hydrogel undergoes *in situ* gelation through chitosan protonation and glycerol monooleate (GMO)-mediated cubic phase transition, significantly prolonging drug retention (Salem et al., 2020). However, the acidic tumor microenvironment selects for aggressive cellular phenotypes with enhanced immune escape capabilities and chemoresistance. Furthermore, acidic conditions induce drug protonation, impairing membrane permeability through the ion trapping effect and significantly compromising chemotherapeutic efficacy (Raghunand et al., 1999; Trebinska-Stryjewska et al., 2020). Ahmed et al. have developed a new pH-responsive drug delivery system for delivering sodium bicarbonate (NaHCO_3_) and doxorubicin (Dox), which improves chemotherapy effects by synchronously responding to and adjusting the pH of the tumor microenvironment (Ahmed et al., 2023) (Figure 2). However, given the dynamic fluctuations of pH within the tumor microenvironment, the long-term efficacy and regulatory mechanisms of pH-responsive systems under sustained acidic conditions require comprehensive validation.

**FIGURE 3:**
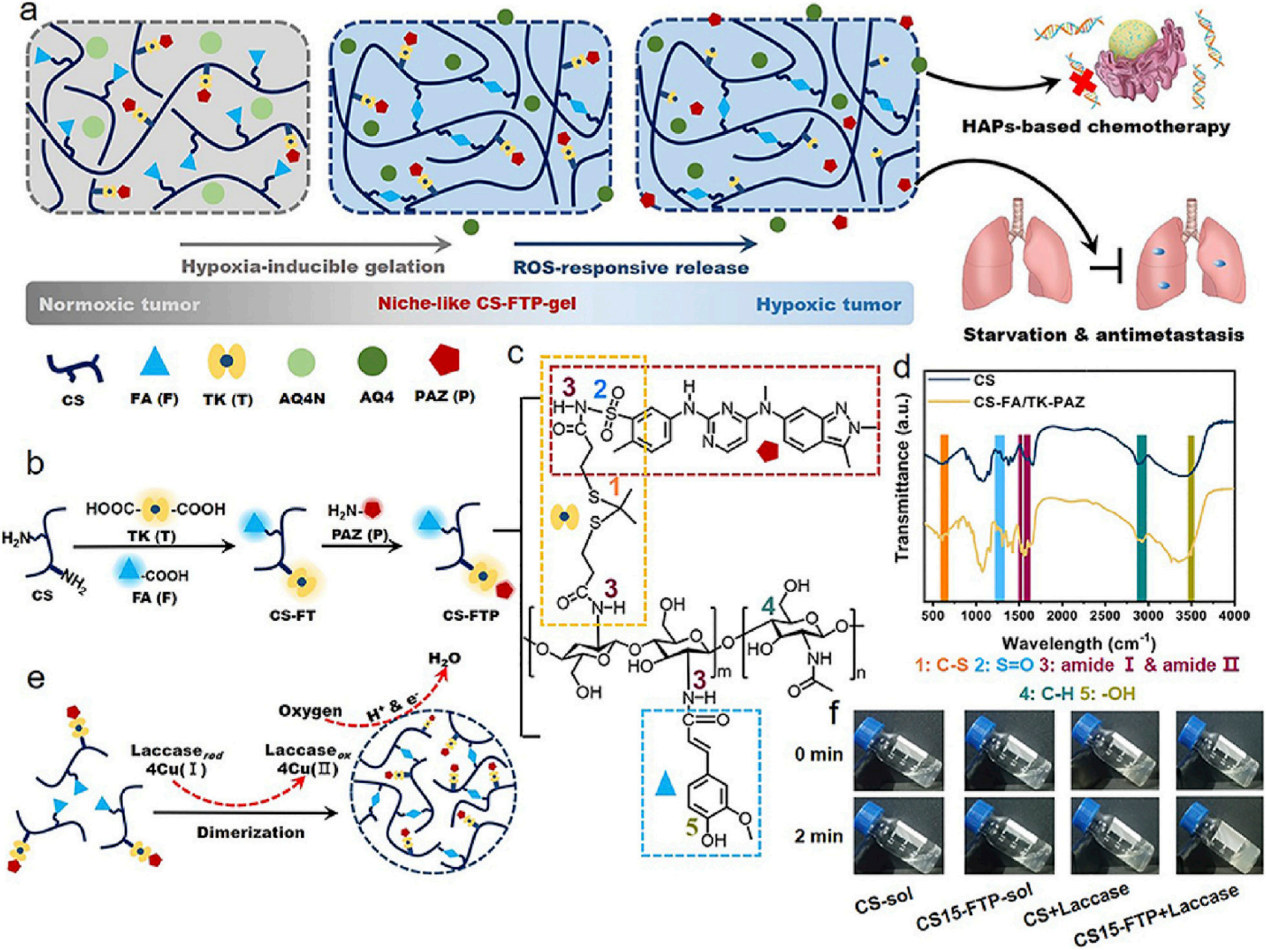
Design CS-FTP-gel. **(a)** Schematic of combination antitumor therapy using niche-like CS-FTP-gel. **(b)** Synthesis of CS-FTP polymer. **(c)** Chemical structure of CS-FTP. Functional moieties conjugated to CS backbone are indicated with dotted boxes of different colors. **(d)** FTIR spectra of CS and CS-FA/TK-PAZ (CSFTP). **(e)** Schematic representation of CS-FTP-gel formation. CS-FTP-gel is formed via laccase-mediated dimerization of FA molecules with oxygen consumption. Laccase catalyzes the four-electron reduction of oxygen to water molecules, resulting in oxidation of FA to form diferulic acid (DiFA) and crosslinking of polymer network. **(f)** Phase transitions of CS solution (CS-sol, 3 wt%) and CS-FTP solution (CS-FTP-sol, 3 wt%) with/without addition of laccase (25 U ml^− 1^). Reprinted with permission from (Chen L.-H. et al., 2023).

Elevated ROS levels characterize the tumor microenvironment. This imbalance not only impacts tumor development and progression but is also closely associated with the regulation of cancer immunity and metabolism. Recent advances in ROS-responsive hydrogels have enabled innovative cancer therapeutic strategies. As shown in the Figure 3, Chen et al. developed a ROS/oxygen dual-responsive chitosan hydrogel (CS-FTP-gel) (Chen S.-X. et al., 2023). It achieves ROS-triggered release of PAZ through a thioketal (TK) linker and enhances tumor hypoxia by laccase-mediated oxygen consumption, synergistically activating the AQ4N chemotherapeutic drug. However, targeting specificity toward bone metastasis and long-term safety still requires further optimization in subsequent research. The balance of redox systems plays a crucial role in tumor development and metastasis. ROS-responsive hydrogels also reduce ROS levels in the environment in response, so long-term dynamic changes need to be taken into account. In addition, there is still controversy about the pro-tumor and anti-tumor effects of ROS at different stages (Glorieux et al., 2024). In the future, achieving a dynamic balance of ROS levels during treatment may be the focus of optimizing such materials.

**FIGURE 4:**
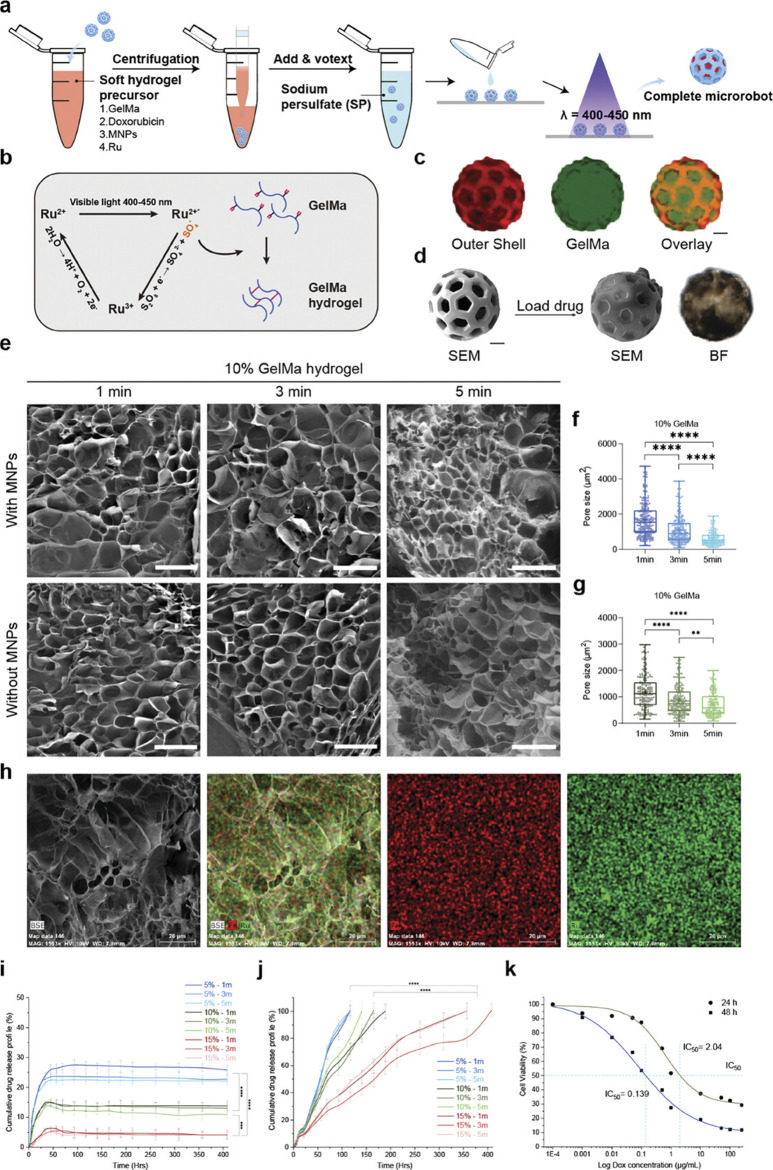
Sustained Stimuli-Responsive Drug Release from Soft Core Hydrogel with Immobilized Therapeutics. **(a)** Overall cargo loading process of ChemoBots. **(b)** Schematic representation of the photocrosslinking process of gelatin methacryloyl (GelMA) using a ruthenium (Ru) sodium persulfate (SPS)-based photoinitiator. **(c)** Fluorescence microscopy images of the hard outer shell of ChemoBots dyed with Eosin (in red) and after loading with FITC-stained hydrogel (in green) and an overlay image to verify the cargo loading protocol. Scale bar, 20 μm. **(d)** SEM image of a ChemoBot after being loaded with GelMA containing Dox and MNPs. Scale bar, 20 μm. **(e)** SEM images showing the morphology of Ru-crosslinked GelMA hydrogels with and without MNPs after exposure to visible light (400–450 nm) for 1, 3, and 5 min, facilitating the covalent crosslinking of free tyrosine and acryl groups (scale bars, 200 µm). **(F,G)** Pore size distribution in 10% w/v GelMA hydrogels post 1, 3, and 5 min of curing, both with and without the incorporation of MNPs. The data indicate a decrease in the average pore size with increasing curing time (n = 3). **(h)** EDX elemental mapping, highlighting the uniform distribution of Ru and Fe, evidencing the homogeneous incorporation of MNPs within the crosslinked GelMA matrix. Scale bar, 20 μm. **(i)** Cumulative release profile of Dox over 400 h in phosphate-buffered saline (PBS), illustrating the initial high burst release rates for 15%, 10%, and 5% drug-immobilized GelMA, which were 6%, 15%, and 23.5%, respectively, within the first 48 h (n = 6). **(j)** Cumulative Dox release profile under tumor microenvironment stimuli, simulating enzymatic activity, demonstrating prolonged and sustained release durations of 117, 165, and 357 h for 5%, 10%, and 15% drug-loaded GelMA, respectively (n = 6). **(k)** Half-maximal inhibitory concentration (IC50) values indicating the doses of the drugs required to inhibit 4T1 cell growth by 50% after 24 and 48 h, providing insights into the therapeutic efficacy of the drug-loaded hydrogel. Reprinted with permission from (Tian et al., 2025).

There are various highly expressed biological enzymes in the breast cancer microenvironment (Jiang et al., 2023; Feng et al., 2025). Enzyme-instructed supramolecular assembly enables fabrication of drug-delivering hydrogels, representing an emerging strategy. There is a research report on the design of a three-enzyme (alkaline phosphatase ALP, carboxylesterase CES, and proteinase K) responsive polypeptide hydrogel (Wu et al., 2021). It forms nanofibers through the self-assembly triggered by ALP and CES, and releases the drug lonidamine (LND) under the action of proteinase K, achieving spatiotemporal regulation of chemotherapy and mitochondrial function disruption. Similarly, the phosphatase-responsive hydrogel system designed by Chen et al. can target the CA IX enzyme on the membrane of hypoxic tumor cells to alleviate the hypoxic environment of the tumor and ultimately enhance the immune system’s ability to kill the tumor (Shi et al., 2024). Furthermore, enzymatic catalysis exhibits high selectivity, specificity, and exceptional catalytic efficiency (Jiang et al., 2025). Recently, Tian et al. developed a novel enzyme-responsive smart hydrogel microrobot (ChemoBots) for anticancer drug delivery to inhibit triple-negative breast cancer (TNBC) tumor growth and reduce its metastasis (Tian et al., 2025). As illustrated in Figure 4, this drug release mechanism is designed according to the tumor microenvironment and is regulated by overexpression of matrix metalloproteinase (MMP2 and MMP9) enzyme activities in TNBC tumors, which triggers degradation of the hydrogel matrix, leading to further controlled release of the drug.

#### 5.1.2 Exogenous stimulus response

**FIGURE 5:**
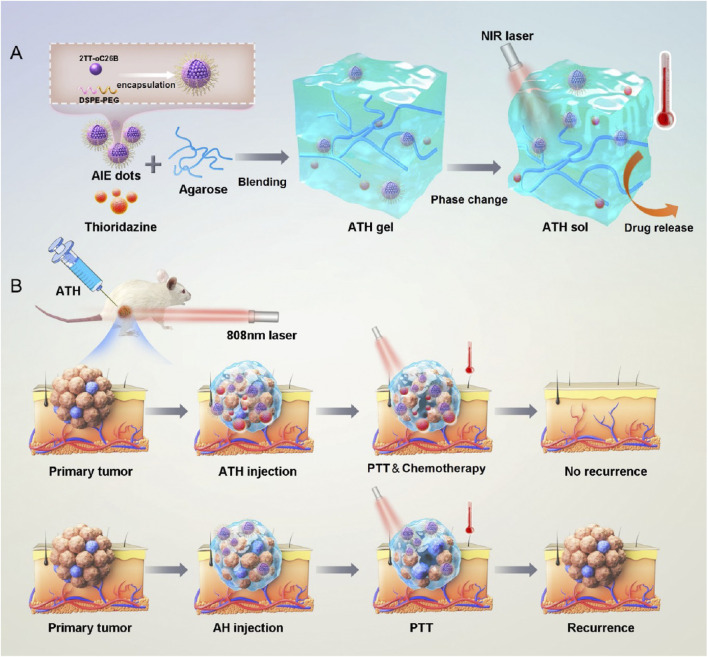
**(a)** Schematic illustration of the preparation of ATH gels and its thermoresponsive sol−gel transition. **(b)** Photothermal-responsive hydrogel triggers combinational drug delivery for killing CSCs and preventing tumor recurrence. Reprinted with permission from (Zhang T. et al., 2024).

Exogenous-responsive hydrogels achieve spatiotemporal control through external stimuli, enabling deep-tissue penetration and synergistic therapies. Wang et al. developed a dual-functional strategy based on a photo-responsive hydrogel for preventing postoperative recurrence and metastasis of TNBC (Wang H. et al., 2021). Composed of a polydopamine-crosslinked collagen/silk fibroin composite, this hydrogel facilitates near-infrared (NIR) light-controlled thrombin release. Under NIR irradiation, the photothermal effect generated by the hydrogel not only triggers thrombin release but also induces peritumoral vascular thrombosis, starving tumors of nutrients. Recently, Zhang et al. developed a photo-responsive hydrogel by co-encapsulating an aggregation-induced emission (AIE) photosensitizer and thioridazine (THZ) within the hydrogel, demonstrating that the AIE photosensitizer-triggered controllable delivery system could enhance THZ-mediated CSC ablation (Figure 5) (Zhang T. et al., 2024). Although light exhibits excellent controllability, it is inevitable that different types of light sources cause damage to normal biological cells. Moreover, the tissue penetrability varies among different light sources, whereas bone metastatic tumor tissues are typically located in deep regions. Optimizing light sources and engineering photoactivated hydrogels for deep-tissue applications remains a critical challenge to reconcile therapeutic efficacy with biosafety.

**FIGURE 6:**
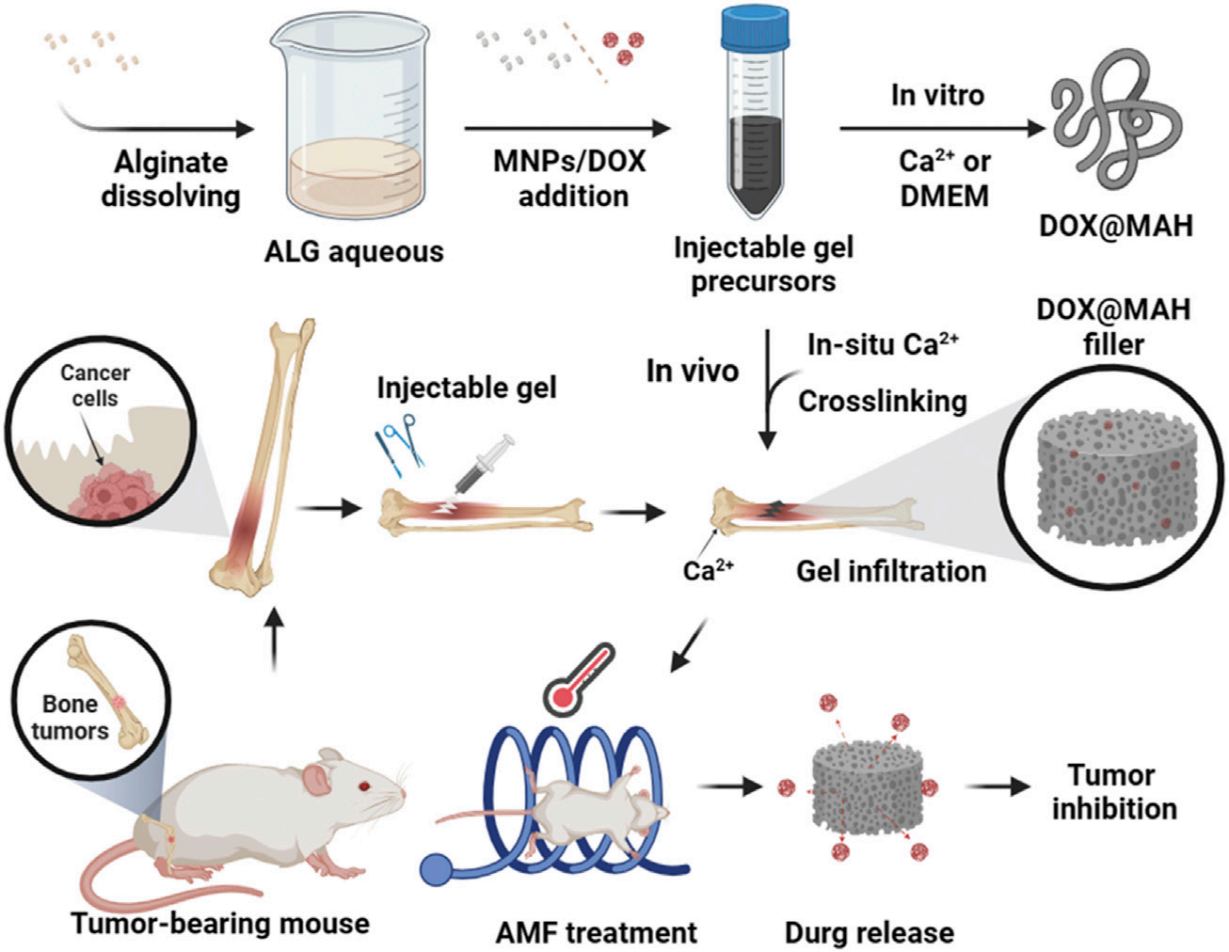
Schematic illustration of Dox@MAH-mediated hyperthermia chemotherapy for bone tumor treatment. Reprinted with permission from (Hu et al., 2024).

The magnetically responsive hydrogel system, as an emerging drug delivery platform, demonstrates significant advantages and broad application prospects. Hu et al. developed a novel injectable magnetically responsive hydrogel-based drug delivery system (DOX@MAH) for the synergistic treatment of bone tumors (Hu et al., 2024). The research team engineered an alginate hydrogel incorporating doxorubicin DOX and magnetic iron oxide nanoparticles, which generates a magnetothermal effect under an alternating magnetic field (AMF). This effect elevates local temperatures above 50°C, directly inducing thermal ablation of tumor cells, while synchronously triggering thermally controlled DOX release, thereby efficiently eliminating tumor cells and suppressing recurrence (Figure 6). In practical applications, how to ensure the safety and convenience of AMF equipment when utilizing such materials also remains a challenge requiring consideration and resolution.

### 5.2 Multifunctional hydrogel platform: Gene-activated therapeutics for precision intervention

The heterogeneity of the BCBM microenvironment and treatment tolerance limit the efficacy of conventional therapies. Recent advances in gene and cell therapies (ATMPs) show promise in reprogramming tumor pathways and activating anti-tumor immunity. However, delivering these biologics faces challenges: rapid degradation, poor targeting, and off-target effects (Ashique et al., 2022; Frederico et al., 2025). Hydrogels, with biocompatible 3D networks, protect biologics and enable sustained release at target sites. Smart stimulus-responsive designs and targeting ligands enhance precision (Su et al., 2024). Critically, multifunctional hydrogel platforms can simultaneously deliver multiple therapies—such as chemotherapy, photothermal therapy, tumor gene silencing, immune reprogramming, and blockade of osteolytic signaling—in a single administration. This synergistic multi-mechanistic approach provides a novel therapeutic strategy to disrupt the BCBM vicious cycle.

**FIGURE 7:**
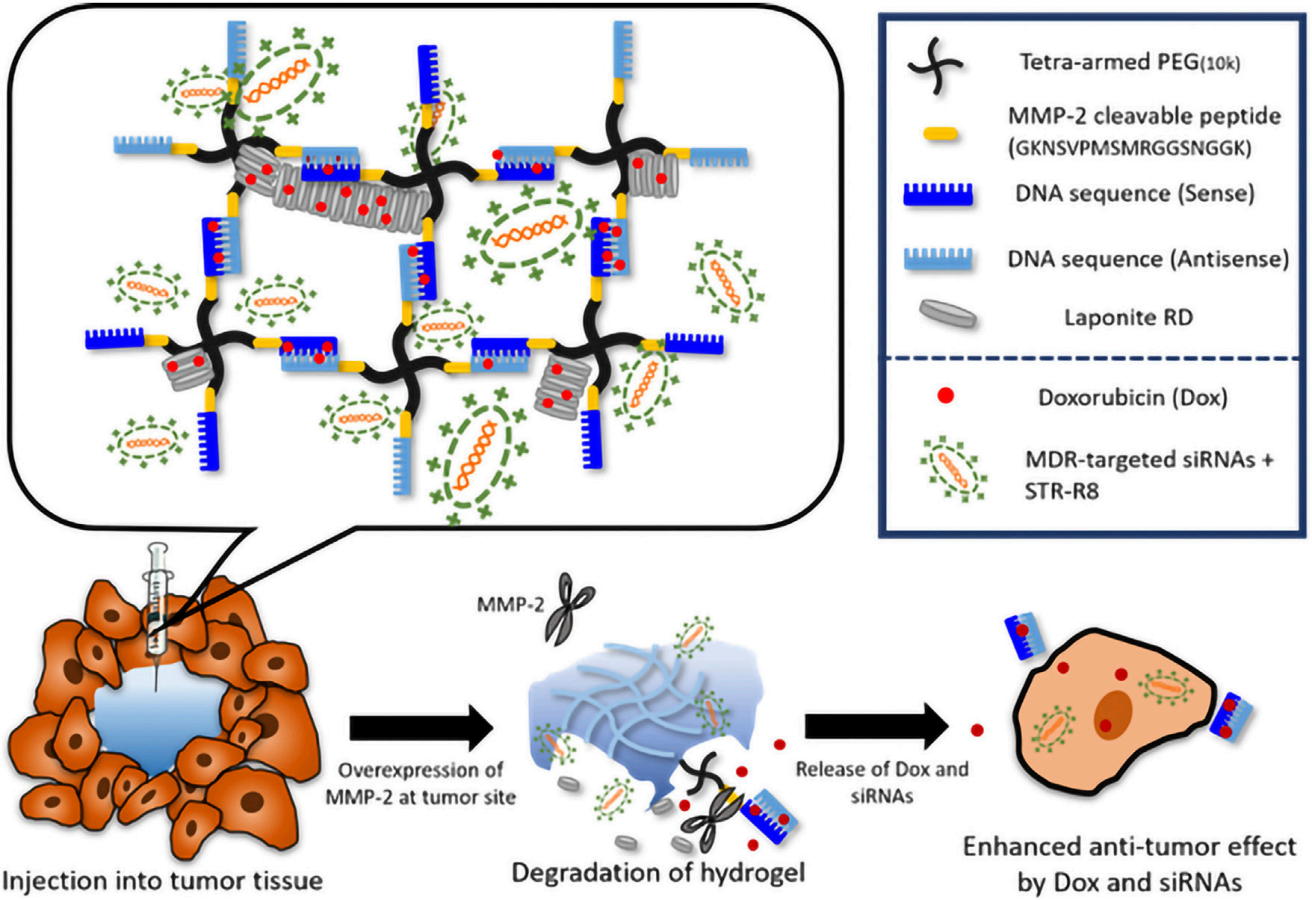
Schematic illustration of the tailor-designed DNA-crosslinked hydrogel network structure and hydrogel degradation mechanism. Triggered by the tumor- specific enzyme (MMP-2), therapeutic cargos are released in multidrug resistant cancer. Reprinted with permission from (Chen L.-H. et al., 2023).

The core value of hydrogel-based ATMP delivery lies in its precise intervention of key tumor-bone interaction pathways. Sophisticated hydrogel carrier design achieves deep integration with molecular mechanisms to suppress tumor proliferation and metastasis. As illustrated in Figure 7, an injectable supramolecular DNA-crosslinked hydrogel was developed for co-delivering the chemotherapeutic drug doxorubicin (Dox) and siRNA targeting multidrug resistance (MDR) to overcome drug resistance in breast cancer (Chen L.-H. et al., 2023). This system enables tumor-specific drug release through enzyme-responsive degradation. The siRNA targeting key resistance-associated genes (P-glycoprotein/Pgp and the anti-apoptotic protein Bcl-2) exhibited enhanced cellular uptake efficiency via incorporation of the cell-penetrating peptide STR-R8, achieving sustained and potent gene silencing in drug-resistant cells (MCF-7/ADR). Furthermore, the cytotoxic efficacy of the co-delivered Dox was significantly amplified following siRNA-mediated suppression of the resistance mechanisms. In a subcutaneous breast tumor model, the hydrogel co-delivery group (Dox + siRNA) achieved 90% tumor volume suppression and displayed the most pronounced apoptotic signaling. Survivin is highly expressed in TNBC and promotes tumor progression by inhibiting apoptosis (Garlapati et al., 2023). To address this, Chen et al. developed a HER2/CD44 dual-targeting hydrogel-integrated nanorobot (ALPR) (Chen J. et al., 2023). This nanorobot combines multiple therapeutic modalities—including gene silencing, mitochondrial disruption, and receptor blockade—demonstrating significant potential to overcome the clinical challenge of BCBM. The ALPR system was constructed by crosslinking trastuzumab (Herceptin) and hyaluronic acid (HA) to form a nanogel shell encapsulating lipid nanoparticle (LPRs) loaded with Survivin siRNA and the pro-apoptotic peptide KLA-R16. This design enables precise tumor targeting and controlled drug release. The dual HER2/CD44 receptor targeting effectively covers major recurrent breast cancer subtypes, addressing tumor cell heterogeneity. Results showed that ALPR reduced survivin expression by 70% in both SKBR-3 and MDA-MB-231 cells. *In vivo* studies demonstrated that a single dose of ALPR achieved over 93% tumor growth inhibition, completely prevented recurrence, and significantly reduced lung metastasis. Although this study did not directly employ a bone metastasis model, the ALPR design exhibits inherent compatibility with the BCBM microenvironment. Precise targeting of the RANKL gene is recognized as an effective strategy to block osteoclast activation and disrupt the vicious cycle of BCBM (Pilard et al., 2025). Yu et al. developed a cancer cell membrane-camouflaged chitosan-polypyrrole nanogel (CH-PPy NG) co-loaded with docetaxel and RANK siRNA (Yu et al., 2024). This platform enables pH-responsive drug release, simultaneously enhancing chemotherapy and silencing the RANK/RANKL pathway, thereby significantly suppressing prostate cancer bone metastasis. Notably, modifying its targeting ligand allows specific enrichment at breast cancer lesions, effectively blocking the “tumor-osteoclast” vicious cycle in breast cancer. Additionally, Yamamoto et al. developed an amphiphilic dextran-dendritic gel (C12-GD-Spe) for efficient delivery of anti-OC-STAMP siRNA, selectively inhibiting pathological bone resorption (Yamamoto et al., 2024). Following local injection, this system sustained siRNA release for over 24 h, achieving up to 70% silencing efficiency without disrupting physiological bone remodeling maintenance. This approach offers a novel strategy for precise bone protection in BCBM. These studies demonstrate that hydrogel carriers, leveraging their advantages in protecting nucleic acid integrity, enhancing tumor targeting, and improving intracellular delivery efficiency, are enabling the transition of genetic interventions from concept to clinical practice.

**FIGURE 8:**
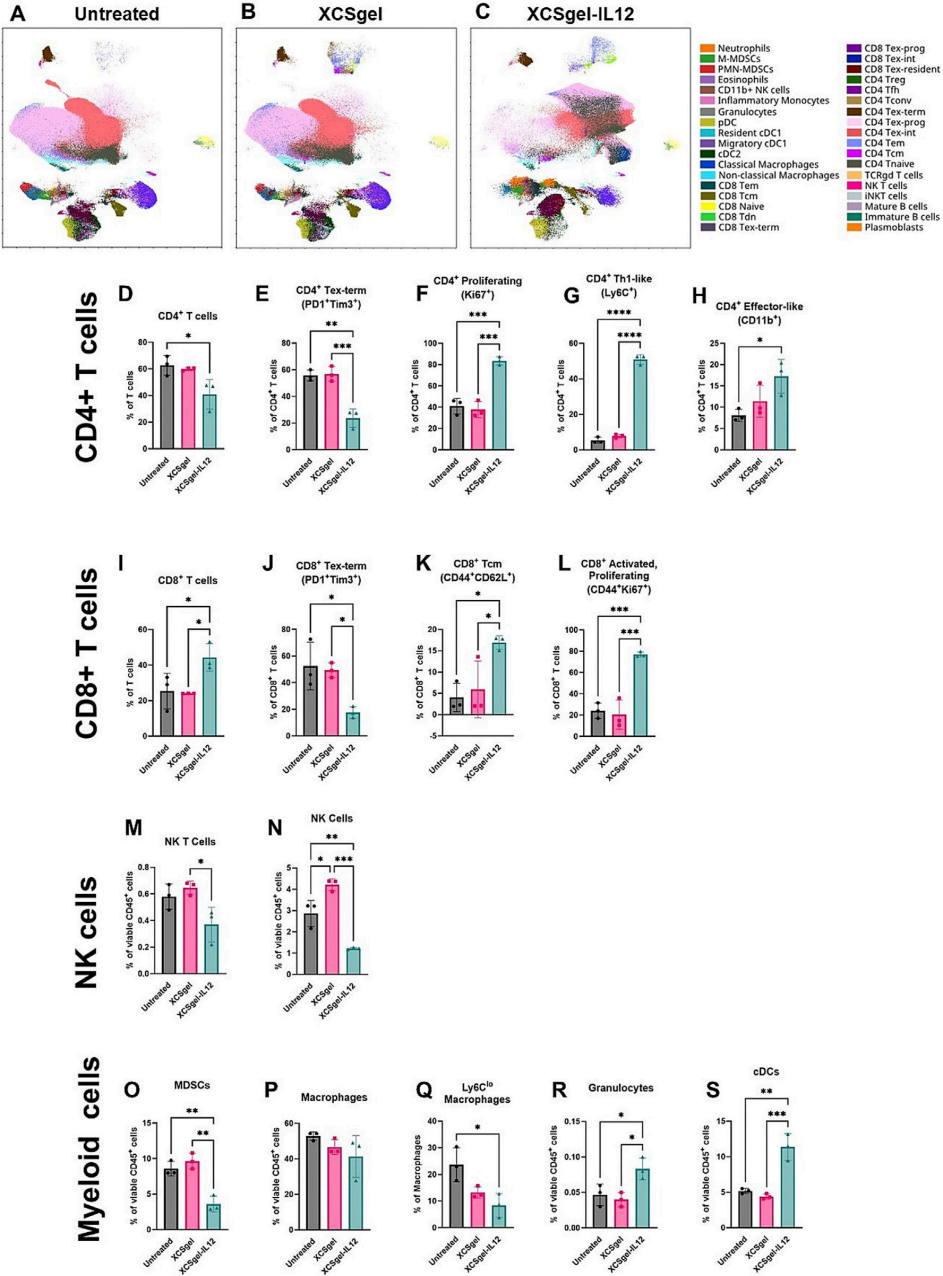
XCSgel-IL12 significantly alters the lymphoid cell compartment. Orthotopic primary E0771 tumors were established 12 days prior to treatment with XCSgel_slow_-IL12_5μg_ or XCSgel_slow._ Seven days after treatment, frequencies and phenotypes of immune cell infiltrates were assessed via spectral flow cytometry. **(a–c)** UMAP visualization of leukocytes populations and phenotypes of **(a)** untreated tumors, **(b)** tumors treated with XCSgel_slow_ alone, **(c)** tumors treated with XCSgel_slow_-IL12_5μg_. (D–X) Frequencies of cell populations and phenotypes. **(d)** CD4^+^ T cell population. For cell frequencies of CD4^+^ T cells, phenotypes include **(e)** terminally exhausted (PD-1 + Tim3+), **(f)** proliferating (Ki67+), **(g)** Th1-like (Ly6C+), **(h)** effector-like (CD11b+). **(i)** CD8^+^ T cell population. For cell frequencies of CD8^+^ T cells, phenotypes include **(j)** terminally exhausted (PD-1 + Tim3+), **(k)** central memory (CD44 + CD62L+), **(l)** activated and proliferating (CD44 + Ki67+). **(m)** NK T cell (CD3 + NK1.1+) frequencies of CD45^+^ leukocytes, **(n)** NK cell (CD3-NK1.1+) frequencies of CD45^+^ leukocytes. Cells within the myeloid cell compartment include **(o)** myeloid derived suppressor cells, **(p)** macrophages, **(q)** Ly6C^lo^ macrophages, **(r)** granulocytes, and **(s)** conventional dendritic cells. Statistical significance was determined using one-way ANOVA and Tukey’s HSD posthoc testing. ^∗^ *P* < 0.05, ^∗∗^ *P* < 0.01, ^∗∗∗^ *P* < 0.001, ^∗∗∗∗^ *P* < 0.0001. *n* = 3 mice per experimental group. Reprinted with permission from (Mantooth et al., 2025).

Breakthrough progress in hydrogel-based ATMP delivery is further demonstrated by its capacity to reverse the immunosuppressive microenvironment in BCBM. Systemic immunotherapy faces dose-limiting toxicity, while conventional intratumoral injection suffers from poor drug retention due to solution extravasation (Albandar et al., 2020). Interleukin-2 (IL-2) plays a critical role in T cell growth and differentiation and was the first approved cancer immunotherapy globally (Im et al., 2024). Yang et al. developed an optimized IL-2 hydrogel delivery platform, ULNPs-cRNAIL-2F@G (Yang K. et al., 2025). This system achieves dual immunomodulation by simultaneously activating CD8^+^ T cells and suppressing regulatory Tregs, significantly enhancing anti-tumor immune responses. Consequently, it effectively inhibits tumor growth while substantially reducing IL-2-associated systemic toxicity. Its excellent safety profile and streamlined preparation process successfully overcome the limitations of conventional IL-2 therapy, expanding its application in tumor immunology with strong clinical translation potential. Interleukin-12 (IL-12) is recognized as one of the most potent antitumor cytokines (Ravichandran et al., 2025). A novel strategy utilizing the viscoelastic hydrogel XGel enables intratumoral retention and sustained release of IL-12 (Mantooth et al., 2025). A single injection effectively reprograms the immunosuppressive tumor microenvironment in TNBC. This system significantly reduces the proportion of exhausted CD8^+^ T cells while increasing activated, proliferating CD8^+^ T cells. The approach not only eradicated 86% of primary tumors but also induced systemic immunity, leading to complete regression of untreated distal tumors in 67% of mice—all without systemic toxicity (Figure 8). This confirms that localized immunoreprogramming can achieve systemic tumor control, offering a potential alternative to systemic cytokine administration. Reprogramming TAMs has emerged as a key therapeutic focus (Li M.-Y. et al., 2024). Liu et al. developed a hyaluronic acid-modified gelatin hydrogel to deliver Siglec-15 shRNA (Liu et al., 2024). Silencing this critical immune checkpoint successfully induced repolarization of M2-type TAMs toward the antitumor M1 phenotype. Combined with photothermal therapy, this approach achieved potent antitumor efficacy. Preclinical studies demonstrated an 80% increase in CD8^+^ T cell infiltration and a 70% reduction in tumor volume within bone metastatic lesions. To counteract chronic tumor inflammation-mediated immunosuppression, the PHOENIX hydrogel scaffold utilizes ROS-responsive programmed release of R848 and anti-OX40 antibody (aOX40) to reverse the immunosuppressive microenvironment in stages (Liang et al., 2024). Initially, R848 preferentially repolarizes MDSCs and M2-TAMs toward an antitumor phenotype. Subsequently, aOX40 expands CD8^+^ T cells while suppressing regulatory T cell (Treg) function, ultimately increasing the CD8^+^/Treg ratio by 15.7-fold. In a TNBC model, the PHOENIX system demonstrated potent inhibition of primary tumor progression and metastatic dissemination. It concurrently established durable antigen-specific immune memory, completely preventing tumor recurrence. Furthermore, this hydrogel platform significantly reduced pro-metastatic factors (IL-6, TGF-β), disrupting the “inflammation-bone destruction-tumor growth” vicious cycle. This approach provides a novel local-systemic immunotherapy paradigm for BCBM. The combined application of gene therapy and adoptive cell therapy (ACT) represents a promising strategy in cancer immunotherapy. A recent study designed the GD-920 bilayer hydrogel scaffold, which innovatively integrates gene therapy (Bim mRNA) with adoptive T cell therapy through a spatiotemporally sequential release mechanis (Lei et al., 2025). Bim mRNA nanocomplexes in the outer layer are released first, inducing immunogenic cell death (ICD) in tumor cells. This releases tumor-associated antigens (TAAs), activating dendritic cells (DCs) and triggering the expansion of antigen-specific CD8^+^ T cells. Subsequently, T cells encapsulated in the inner layer are released, enabling high-density infiltration and targeted elimination of residual tumor cells. This creates a closed-loop immune activation cycle. Experimental data revealed that GD-920 combination therapy significantly enhanced tumor suppression compared to monotherapies, achieving 90% clearance of primary tumors in mouse models. This approach provides a novel integrated therapeutic paradigm to address the challenges of immunologically “cold” tumors and postoperative recurrence in BCBM.

In summary, ATMP delivery mediated by multifunctional smart hydrogel systems significantly inhibits BCBM progression and reverses immunosuppression by targeting key molecular pathways (Table 2). This approach overcomes the inherent limitations of monotherapies through synergistic therapeutic mechanisms, establishing a novel therapeutic paradigm for optimized efficacy. The latest combination of hydrogel carriers with naked plasmids shows that it can significantly prolong the in - vivo action time of the delivered genes, further improve the transfection efficiency while avoiding the risks associated with viral vectors (Lu et al., 2024). Future studies could further focus on integrating naked plasmid-based gene-activated materials, Leveraging their low immunogenicity and compatibility with hydrogel carriers, these systems enable enhanced gene delivery efficiency (Krasilnikova et al., 2023; Wallen et al., 2023). These superior outcomes create the basis for shifting the bone microenvironment from destruction to regeneration. The following section will examine strategies for achieving functional bone reconstruction based on this foundation.

**TABLE 2:** Multifunctional hydrogel platforms for BCBM therapy: gene delivery and immune microenvironment reprogramming strategies.

Therapeutics focus	Therapeutic cargo	Core mechanism of action	Ref.
Gene delivery	Doxorubicin anti-Pgp/Bcl-2 siRNA	siRNA-mediated silencing of drug-resistance genes → Chemosensitization	Chen et al. (2023)
	anti-Survivin siRNA KLA-R16 peptide	Gene silencing (Survivin) and Mitochondrial disruption → Synergistic apoptosis induction	Chen et al. (2023)
	Docetaxel anti-RANK siRNA	RANK/RANKL pathway blockade → Disruption of “tumor-osteoclast” vicious cycle	Yu et al. (2024)
	anti-OC-STAMP siRNA	Targeted suppression of osteoclast fusion → Pathological bone resorption inhibition	Yamamoto et al. (2024)
	anti-Siglec-15 shRNA	Immune checkpoint silencing → Repolarization of M2-TAMs to antitumor M1 phenotype	Liu et al. (2024)
Immune reprogramming	Optimized IL-2 (circRNA-encoded)	Selective CD8^+^ T cell activation and Treg suppression → Enhanced antitumor immunity	Yang K. et al. (2025)
	IL-12 cytokine	Reprogramming of immunosuppressive TME → Systemic antitumor immunity activation	Mantooth et al. (2025)
	R848 (TLR7/8 agonist) + α-OX40 Ab	Phase 1: Myeloid cell reprogramming → Phase 2: CD8^+^ T cell expansion/Treg inhibition	Liang et al. (2024)
	Bim mRNA + Adoptive T cells	Outer layer: ICD induction → Antigen presentation → Inner layer: T cell-mediated tumor clearance	Lei et al. (2025)

### 5.3 Hydrogel-mediated remodeling of the bone microenvironment

Following successful suppression of tumor progression, the core goal of bone microenvironment management shifts to structural bone regeneration. BCBM-induced osteolytic destruction leads to progressive bone defects, pathologically characterized by aberrant osteoclast activation and impaired osteogenesis-mineralization balance (Tang et al., 2020; Thilakan et al., 2025). Conventional bone repair materials exhibit low repair efficiency due to their inability to reverse the immunosuppressive microenvironment. Hydrogels have emerged as an ideal platform for achieving “immune remodeling-bone regeneration” through their biomimetic ECM structure, mechanical signaling capability, and multi-payload integration capacity (Xue P. et al., 2025). This dual-functional intervention establishes an innovative therapeutic paradigm for BCBM management, representing a key direction for metastatic microenvironment remodeling and functional bone reconstruction (Table 3).

**TABLE 3:** Multifunctional hydrogel platforms for bone repair.

Therapeutic focus	Hydrogel system	Core mechanism of action	Ref.
A. Activating Osteogenic-Mineralization Cascade			
Three-dimensional bone trabecula bionic structure	the Freeform Reversible Embedding of Suspended Hydrogels (FRESH)	Mimicking the physiological structure of trabecular bone → Promoting the migration of osteoblasts and the ingrowth of blood vessels	Ovejero et al. (2019) Kontakis et al. (2025)
Biomimetic mineralization induction	Degradable ion-doped hydrogel (Ca^2+^/Mg^2+^/PO_4_^3-^)	Gradient ion release → Activates CaSR receptor → Enhances hydroxyapatite deposition	Yao et al. (2024) Chiu et al. (2023)
	Thermosensitive hydrogel of carboxylated carbon spheres with surface modification	NIR photothermal ablation of tumors + electrostatic adsorption of Ca^2+^ to induce heterogeneous nucleation	Wei et al. (2022)
Mechanical programming microenvironment	stiffness gradients (5–50 kPa), microtopographic arrays, magnetically driven dynamic stress	Triggers YAP-RhoA/ROCK feedback loop → Drives MSC osteogenic differentiation	Xue P. et al. (2025)
B. Immune remodeling-osteogenesis synergy			
Targeted blockade of osteoclastogenic signals	Siglec-15-targeted hydrogel (shRNA@BG)	Silencing Siglec-15 → Inhibiting osteoclast genes (c-Fos/TRAP) + Release of Ca^2+^/Si^+^ to promote osteogenic genes (RUNX2)	Liu et al. (2024)
Macrophage phenotypic remodeling	IL-4 delivery hydrogel (Ca-GG + IL-4)	Induce M2 polarization → Increase TGF-β1 → Activate the TGF-β1/Smad pathway in BMSCs	Zhang et al. (2020)
Vascular-osteogenic temporal coupling	Growth factor cascade hydrogel (VEGF/BMP - 2)	Rapid release of VEGF (vascularization) → Sustained release of BMP - 2 (osteogenesis) → Mimicking natural healing	Cao et al. (2024)
Stem cell niche construction	Acid-responsive hydrogel (SDF-1α/Arg-CD)	Acidic microenvironment triggers Release of SDF - 1α Recruitment of stem cells Generation of NO → Activation of NO/cGMP to promote angiogenesis	Xiao et al. (2025)

**FIGURE 9:**
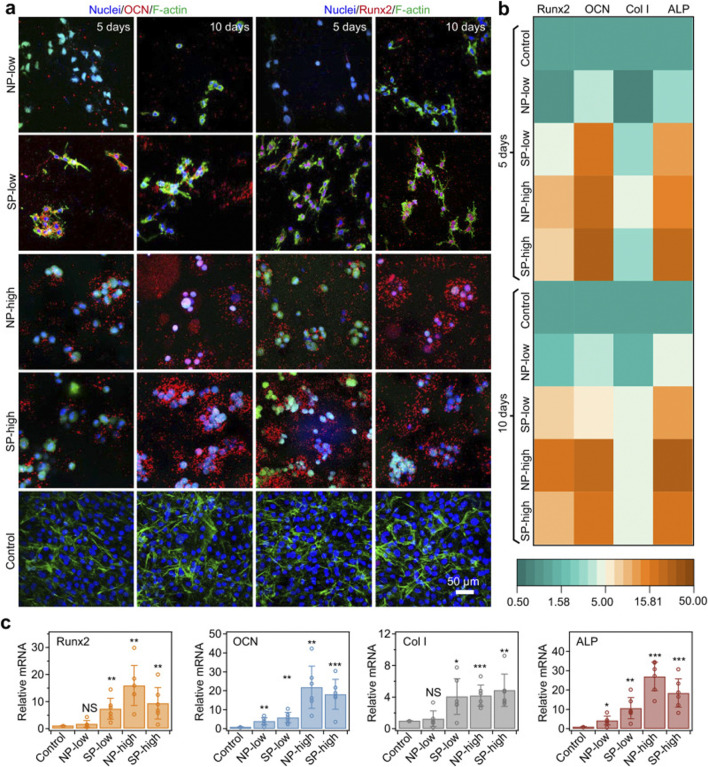
Osteodifferentiation of encapsulated cells driven by consistent mechanical cues of rigid shells. **(a)** MC3T3-E1 cells in different hydrogels identified by immunostaining of molecular markers (OCN or Runx2, shown in red) and F-actin (phalloidin, shown in green) after 5 and 10 days of culture. MC3T3-E1 cells on the cell culture wells served as the control group. OCN and Runx2, specific markers for osteogenic differentiation, were upregulated in SP-low, NP-high and SP-high hydrogels. Cell nuclei were stained with DAPI (blue). A space of 318 μm × 318 μm × 100 μm in each sample was scanned layer by layer, and the images were projected onto the z-axis to show immunostaining. **(b)** Heatmap illustrating mRNA expression levels corresponding to osteogenesis genes (Runx2, OCN, Col I and ALP) for cells in different hydrogels or cell culture wells (Control) after 5 and 10 days of culture. The intensity represents the expression relative to the control group. **(c)** Summary of relative mRNA expression levels of osteogenesis-related genes (Runx2, OCN, Col I, and ALP) in cells cultured in different hydrogels or cell culture wells (Control) after 10 days. The expression levels of these genes were normalized to those of the control group. Values represent the mean ± standard deviation (n = 6 independent experiments). The p-values for the comparisons between the control group and the NP-low, SP-low, NP-high, and SP-high groups are as follows: for Runx2, 0.2938, 0.0048, 0.0012, and 0.0098, respectively; for OCN, 0.0068, 0.0025, 0.0019, and 0.0007, respectively; for Col I, 0.5670, 0.0130, 0.0003, and 0.0020, respectively; and for ALP, 0.0127, 0.0028, 0.00001, and 0.0003, respectively. Statistical significance between different groups and the control group was assessed using two-tailed Student’s t-test. *p < 0.05; **p < 0.01; ***p < 0.001; NS: not significant. Reprinted with permission from (Xue B. et al., 2025).

**FIGURE 10:**
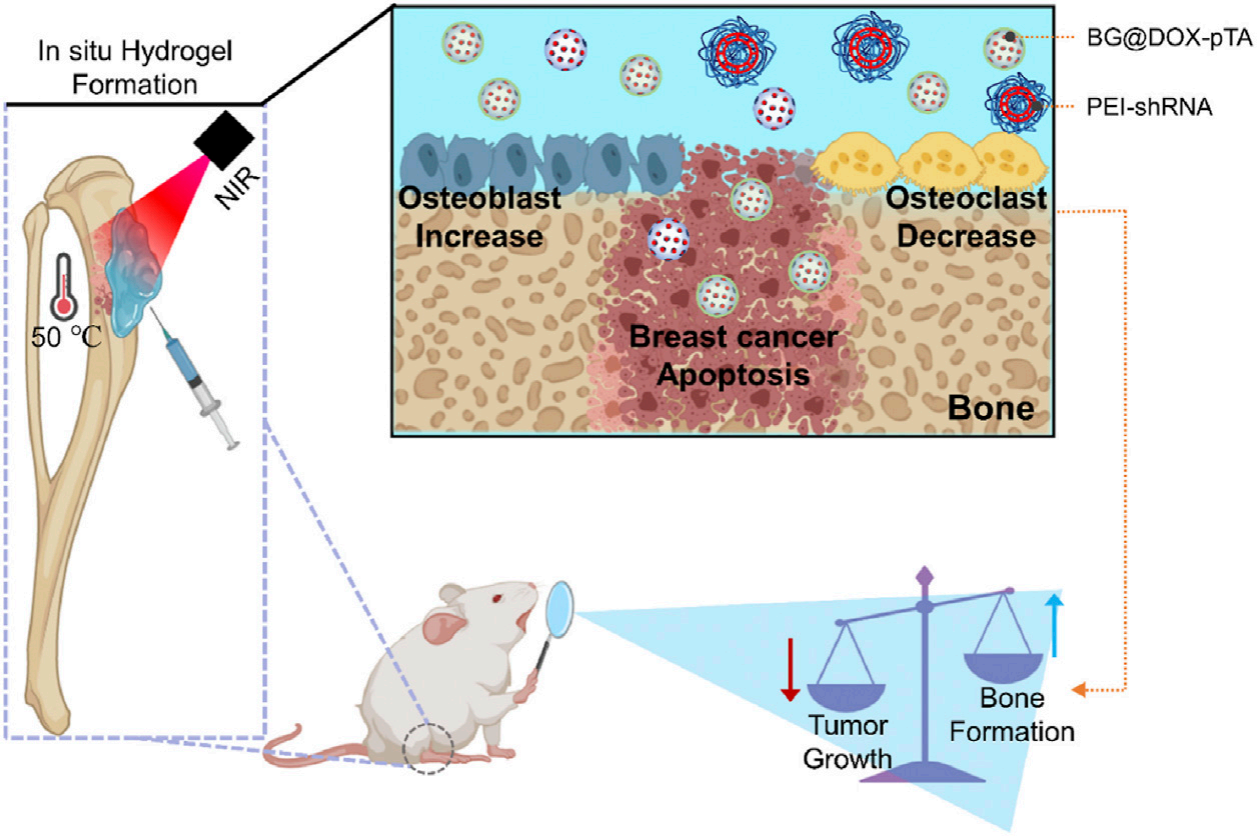
Siglec-15 Targeting Integrated Bioactive Glasses Hydrogel for Treatment of Breast Cancer Bone Metastasis. The hydrogel, composed of a tannic acid/Fe3+ coated doxorubicin-loaded BG particles (BG@DOX-pTA), PEI-Siglec-15 shRNA complexes, and sodium alginate (SA), is designed to integrate photothermal chemotherapy, immunotherapy, and bone repair at the tumor site. Upon injection and subsequent laser irradiation, the hydrogel undergoes *in situ* gelation, generating a localized photothermal-chemotherapy effect that induces immunogenic cell death of cancer cells, while the PEI-shRNA specifically silences Siglec-15, modulating the tumor microenvironment to inhibit osteoclast activity, thereby normalizing bone homeostasis. Ultimately, the dual effects can be achieved to inhibit the tumor growth and normalize bone homeostatic dysregulation in breast cancer bone metastasis. Reprinted with permission from (Liu et al., 2024).

**FIGURE 11:**
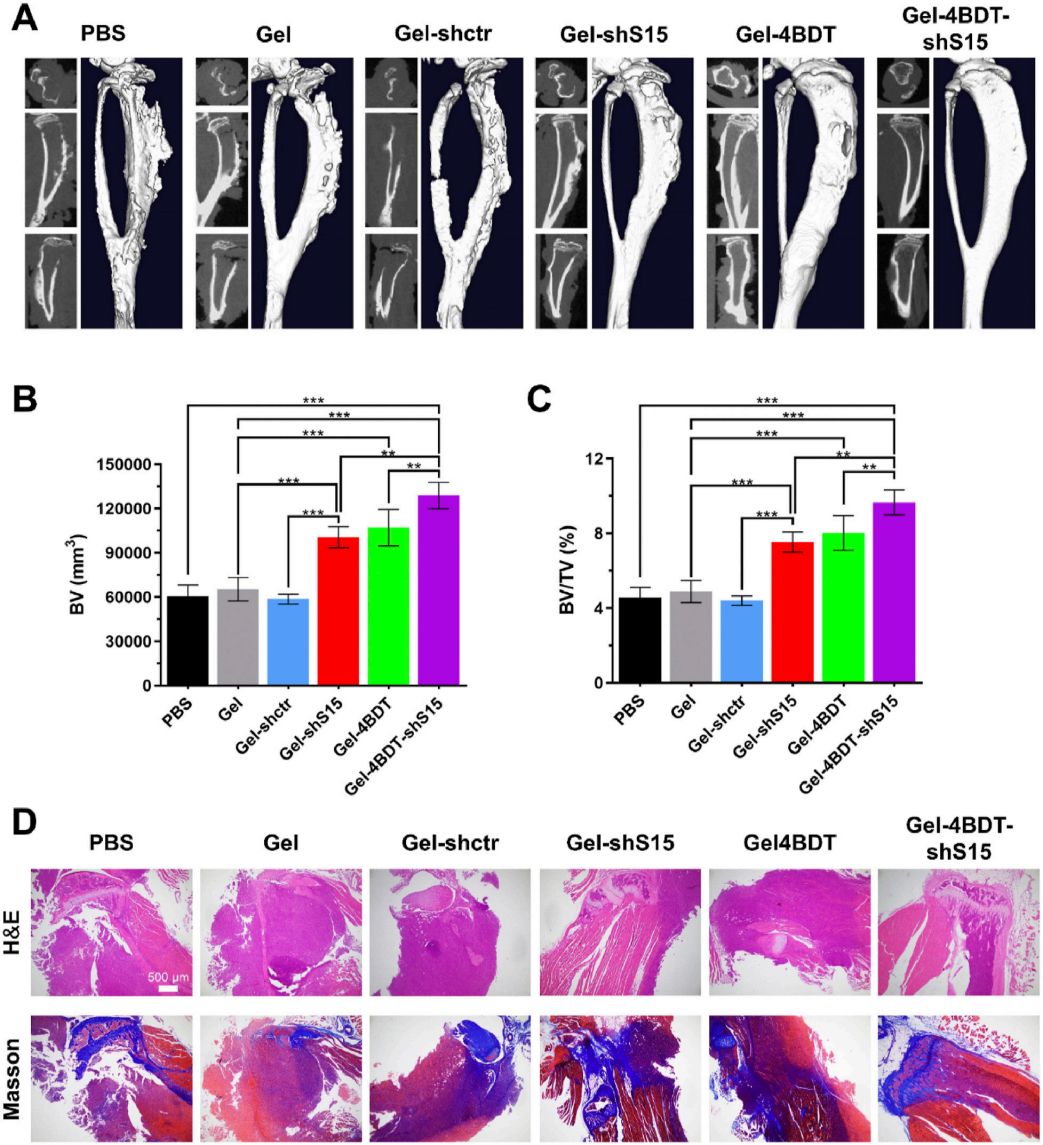
Siglec-15 targeting BG hydrogel inhibits tumor-induced osteolysis and Restores bone homeostasis. **(A)** Micro-CT images of isolated tibias from mice after a 14-day treatment period. **(B,C)** Quantitative analysis of bone volume (BV) and the bone volume/tissue volume ratio (BV/TV). **(D)** Representative H&E and Masson’s trichrome staining of tibias from all treatment groups. Data are presented as means ± SD (n = 3). Statistical significance was calculated via one-way ANOVA. *p < 0.01, ***p < 0.001. Reprinted with permission from (Liu et al., 2024).

Hydrogels precisely regulate the osteogenic cascade through their physicochemical properties. Their three-dimensional porous network adequately simulates the physiological structure of bone trabeculae, significantly enhancing osteoblast migration efficiency and vascularization capacity (Bellani et al., 2021; Zhou J. et al., 2024; Kontakis et al., 2025). By releasing loaded bioactive ions, they activate the CaSR receptor to upregulate calmodulin expression in osteoblasts, promoting hydroxyapatite crystal deposition (Ovejero et al., 2019; Chiu et al., 2023). Synergistic enzymatic mineralization further enhances their mineralization capability (Guo et al., 2022). Postoperative bone defects following bone tumor resection present a significant clinical challenge. To address this, Wei et al. developed an injectable composite hydrogel for bone tumor therapy and regenerative repair of bone defects (Wei et al., 2022). This thermosensitive, injectable hydrogel system undergoes rapid gelation at physiological temperature to conformally fill bone defects of arbitrary geometry. Under 808 nm NIR laser irradiation, it effectively eradicates tumor cells. Simultaneously, carboxylated carbon spheres modified on its surface electrostatically adsorb Ca^2+^ to induce heterogeneous nucleation, facilitating apatite deposition and further promoting bone tissue regeneration. Furthermore, recent research has focused on optimizing mechanotransduction to achieve osteogenic differentiation. Xue et al. developed a multiscale programmable GelMA hydrogel (Xue B. et al., 2025). This system synergistically integrates triple mechanical cues—stiffness gradients (5–50 kPa), microtopographic arrays, and magnetically driven dynamic stress—to activate the YAP-RhoA/ROCK positive feedback loop, significantly promoting osteogenic differentiation of MSCs (Figure 9). This approach provides novel insights for bone repair in advanced BCBM cases, although the long-term maintenance mechanism of mechanical signals *in vivo* requires further exploration. In the BCBM microenvironment, tumor-mediated aberrant osteoclast activation is the core driver of bone destruction, while the immunosuppressive microenvironment further inhibits osteogenic repair. Hydrogels, as efficient delivery vehicles, precisely target the lesion area, offering an innovative strategy to address this challenge by blocking osteolysis, remodeling the bone microenvironment, and synergistically activating osteogenesis. Given the pivotal role of the RANKL signaling axis in breast cancer bone metastasis, precise genetic intervention can effectively block osteoclastogenesis. For instance, Liu et al. developed an integrated bioactive glass (BG) hydrogel targeting Siglec-15, which delivers shRNA to silence Siglec-15 expression in the tumor microenvironment (Figure 10) (Liu et al., 2024). This effectively blocks RANKL-induced expression of key osteoclast genes (c-Fos, TRAP, Cathepsin K), thereby inhibiting osteoclast differentiation (>70% reduction in RAW264.7 cell osteoclast formation). Concurrently, it remodels the immunosuppressive microenvironment by increasing intratumoral CD8^+^ T-cell infiltration. BG-released Ca^2+^/Si^+^/Fe^3+^ ions promote the expression of osteogenic genes (RUNX2, ALP, OPN), further enhancing osteogenic repair (Figure 11). Combined with photothermal-chemotherapy for tumor suppression, this strategy significantly inhibited breast cancer bone metastasis progression and effectively repaired osteolytic lesions in mouse models, providing a novel multifunctional therapeutic platform paradigm to disrupt the “tumor-bone destruction” vicious cycle. Additionally, the M1 macrophage-dominated pro-inflammatory environment in BCBM exacerbates bone destruction (Liu B. et al., 2023). Phenotypic reprogramming of immune cells can effectively reverse the osteolytic microenvironment. An IL-4-loaded calcium-enriched gellan gum hydrogel (Ca-GG + IL-4) locally sustains IL-4 release to polarize macrophages towards the M2 phenotype (Zhang et al., 2020; Xiao et al., 2025) This increases TGF-β1 secretion, activating the TGF-β1/Smad pathway in BMSCs, significantly promoting osteogenic differentiation and inhibiting apoptosis. In rat bone defect models, this hydrogel increased new bone volume by 3.46-fold, providing a novel immunomodulatory approach for bone repair in BCBM.

**FIGURE 12:**
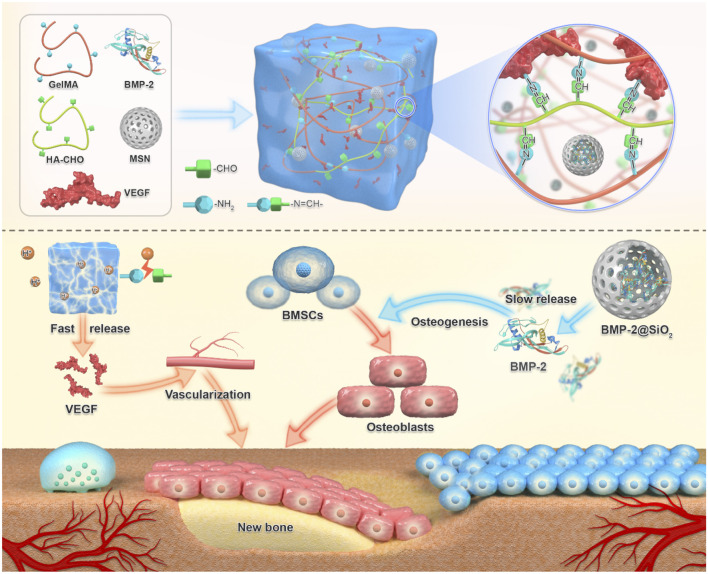
Schematic diagram of BVHG composite hydrogel promoting angiogenesis and enhancing osteogenesis. The composite hydrogel exhibited the capability to promptly release VEGF in the initial response to the acidic bone microenvironment, thereby facilitating early angiogenesis. Additionally, it demonstrated sustained release of BMP-2 over an extended period, thereby promoting osteoinductive characteristics (Cao et al., 2024).

Hydrogels achieve functional bone regeneration by establishing stem cell niches and vascularization networks. Recruitment of endogenous stem cells to bone defect sites is an effective strategy for *in situ* bone regeneration. Xiao et al. developed an acid-triggered bifunctional hydrogel platform (HG-AA1:1-SDF-1α) that intelligently responds to the acidic microenvironment (Xiao et al., 2025). This system continuously releases SDF-1α to recruit endogenous stem cells and generates NO through Arg-CD metabolism, further activating the NO/cGMP signaling pathway to promote angiogenesis, thereby achieving “coupled osteogenesis and angiogenesis”. As shown in Figure 12, a novel composite hydrogel successfully establishes a microenvironment conducive to vascularization and osteogenesis through the cascade delivery of vascular endothelial growth factor (VEGF) and bone morphogenetic protein-2 (BMP-2), significantly enhancing bone regeneration efficacy (Cao et al., 2024). By designing rapid VEGF release and sustained slow BMP-2 release, this hydrogel system mimics the stage-specific growth factor regulation during natural bone healing, ensuring the orderly progression of angiogenesis and osteogenesis. The hydrogel system overcomes the mechanical collapse caused by premature degradation of traditional materials and the foreign body reaction caused by long-term retention through its dynamically adjustable mechanical properties. For instance, Yao et al. developed an injectable dual-ion doped composite hydrogel (SOH1(CP)1) whose degradation cycle precisely matches the bone regeneration process (Yao et al., 2024). Building upon synergistic photothermal-chemotherapy for tumor suppression, it significantly promotes bone repair via gradient release of Ca^2+^/Mg^2+^. This system provides mechanical support in the initial phase (0–4 weeks) and progressively releases ions (Ca^2+^, Mg^2+^, PO_4_^3-^) along with sericin degradation products in the later phase (4–12 weeks), mimicking the natural bone regeneration microenvironment, offering potential for local control and functional recovery of BCBM lesions.

## 6 Conclusion and prospect

Standardized therapeutic strategies for BCBM are currently lacking. The existing intervention measures are mostly symptomatic supportive treatment, focusing on analgesic management, risk control of bone-related events, and tumor progression inhibition. Although systemic treatment occupies a fundamental position in the comprehensive management of BCBM, insufficient local control efficacy and risk of pathological recurrence are still key scientific issues in clinical practice. Local targeting strategies developed based on bone microenvironment characteristics (such as bone-oriented radionuclide therapy, bisphosphonate drugs, and biotargeted preparations) are gradually attracting attention. However, the traditional drug delivery mode is limited by the low permeability efficiency of the blood-bone barrier and the short residency period of drug bone tissue, which often leads to the dual dilemma of increased systemic exposure toxicity and insufficient effective concentration of lesion target areas. In this context, biomaterial-driven local sustained release delivery systems provide innovative directions to resolve this contradiction, in which an injectable hydrogel platform with bone tissue adaptation characteristics demonstrates unique therapeutic advantages.

Hydrogel technology has advanced from conventional drug delivery systems to stimuli-responsive platforms enabling precise multimodal therapeutics. By integrating multi-stimuli-responsive mechanisms, gene-editing tools, 3D bioprinting frameworks, and AI-driven predictive models, this technology enables real-time sensing and closed-loop regulation of dynamic bone microenvironmental cues, such as cytokine gradients, hypoxic states, and mechanical stress. Concurrently, its dual functionality in inducing bone matrix regeneration and vascularization positions hydrogel-based systems as an innovative strategy for synergistic treatment of breast cancer bone metastasis (Ge et al., 2025). Simultaneously, through modulation of the bone microenvironment and integration of bioactive molecules, these intelligent hydrogel systems not only exhibit high-efficiency antitumor efficacy but also possess robust bone-repairing properties, serving as a highly impactful novel tool for postoperative bone defect restoration in breast cancer bone metastasis (Mansouri Moghaddam et al., 2025). Future studies should integrate organoid models with intelligent hydrogels to precisely target key nodes in the “tumor colonization–osteoclast activation–immune escape” malignant cascade. Among them, the biomimetic organoid model based on hydrogel construction, with its three-dimensional pathological ecological niche reconstruction ability (such as decellularized bone matrix integration, co-culture of patient-derived tumor cells), has become the core research tool for analyzing the vicious cycle of “tumor colonization - osteoclast activation - immune escape”, providing an unprecedented research platform for revealing the colonization, dormancy activation, and drug resistance evolution of breast cancer cells (Esmaeili et al., 2021; Zuo et al., 2022). For example, Ji et al. successfully developed a 3D hydrogel-bone organoid coculture system, which mimics the pre-metastatic niche (PMN), to elucidate the interactions between bone-resident cells and metastatic tumor cells under the influence of primary cancer (Ji et al., 2023). Further studies revealed that the mechanical properties of hydrogels (e.g., stiffness, porosity) dynamically regulate phenotypic switching in tumor cells. For instance, by constructing hydrogel models mimicking the mechanical properties of bone marrow, researchers demonstrated how mechanical stimuli (perfusion and compression) modulate tumor cell proliferation, protein expression, and three-dimensional growth, thereby providing standardized tools for investigating bone metastasis mechanisms (Curtis et al., 2020).

Despite the unique advantages demonstrated by composite hydrogel systems in the localized treatment of breast cancer bone metastasis, their clinical translation remains hindered by multiple technical bottlenecks. First, while many hydrogel materials have shown negligible biotoxicity *in vitro* and *in vivo* studies, most evaluations rely on lower-level animal models, and their biosafety in humans requires further validation (Li Y. et al., 2024). Secondly, how to achieve higher component load while maintaining the injectability of hydrogels is still the core issue in the future design of such materials (Li et al., 2025). More critically, existing drug-controlled release strategies exhibit spatiotemporal mismatches with the dynamic evolution of bone metastatic lesions. Current reported responsive release systems struggle to adapt to multidimensional biological signal changes in the metastatic microenvironment. Concurrently, studies reveal that incorporating hydroxyapatite nanocrystals reduces the hydrogel’s shear-thinning performance, impairing its infiltration and diffusion capabilities within dense bone tissues. Furthermore, the physical barrier formed by the highly mineralized bone matrix imposes pressure-dependent delivery efficiency limitations on hydrogel intralesional injection (Tan et al., 2022). Overcoming these barriers to achieve precise drug delivery to deep-seated bone metastases will be a key future research focus. The mechanisms underlying multicomponent synergistic therapy remain insufficiently understood. When hydrogels are co-loaded with bisphosphonates, anticancer drugs, and bone-repairing factors, the pharmacokinetic interactions among these components during the bone resorption-release cycle—and their potential impact on the therapeutic window—urgently require systematic evaluation. At present, the hydrogel drug delivery platform is still in the early stage of technological development, and its transformation to large-scale industrial-level production faces significant challenges, including key bottlenecks such as process standardization, batch stability control, and cost-effectiveness optimization (Rezakhani et al., 2024). At the same time, robust regulatory frameworks for ATMPs remain a paramount challenge globally (Sanchez-Guijo et al., 2024). The heterogeneous characteristics of bone metastasis in breast cancer determine that the treatment plan needs to be highly individualized. In order to achieve the desired therapeutic effect, this demand will undoubtedly further increase production costs and increase the economic burden on patients. How to achieve a balance between therapeutic effect and cost may become a new problem that urgently needs to be solved in the research and development of future intelligent responsive hydrogels. The solution to this challenge may require interdisciplinary collaboration, covering innovations and cooperation in multiple fields such as materials science, oncology, and health economics.

While significant translational challenges remain for the clinical implementation of current intelligent hydrogel-based drug delivery systems, recent advancements have notably transcended the limitations of unidirectional therapeutic delivery, evolving toward a multidimensional coordination paradigm encompassing precision-targeted therapy, immunomodulation, and functional osteoregeneration. It can be predicted that driven by interdisciplinary cooperation, the advanced intelligent hydrogel system is likely to achieve a significant leap from basic research to clinical transformation, opening a promising new chapter for the treatment of BCBM.
